# Supplementary figures and images for: Genome-wide maps of distal gene regulatory enhancers active in the human placenta
Source: PLoS One. 2018 Dec 27;13(12):e0209611. doi: 10.1371/journal.pone.0209611 (PMC6320013; doi:10.1371/journal.pone.0209611)

**A** Enhancer vs. Non-enhancer

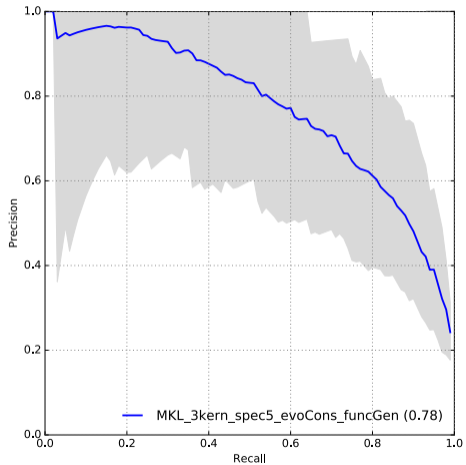

**B** Placenta vs. Other Tissues

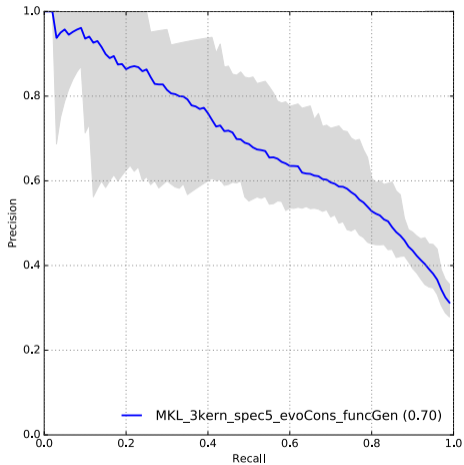

Supplement: S1 Fig — (A) Precision-recall (PR) curves for the classifiers trained to distinguish enhancers from non-enhancers (Step 1) and (B) placental enhancers from enhancers active in other tissues (Step 2). Both perform significantly better than expected by chance with areas under the PR curve (AUC) of 0.78 and 0.70 respectively. The shaded region represents the performance range observed over the 10 cross validation runs. (PDF) [file pone.0209611.s001.pdf]
